# CT and MRI radiomics of bone and soft-tissue sarcomas: an updated systematic review of reproducibility and validation strategies

**DOI:** 10.1186/s13244-024-01614-x

**Published:** 2024-02-27

**Authors:** Salvatore Gitto, Renato Cuocolo, Merel Huisman, Carmelo Messina, Domenico Albano, Patrick Omoumi, Elmar Kotter, Mario Maas, Peter Van Ooijen, Luca Maria Sconfienza

**Affiliations:** 1 Dipartimento di Scienze Biomediche per la Salute, Università degli Studi di Milano, Milan, Italy; 2 IRCCS Istituto Ortopedico Galeazzi, Milan, Italy; 3 Department of Medicine, Surgery and Dentistry, University of Salerno, Baronissi, Italy; 4 Radboud University Medical Center, Department of Radiology and Nuclear Medicine, Nijmegen, The Netherlands; 5 Dipartimento di Scienze Biomediche, Chirurgiche ed Odontoiatriche, Università degli Studi di Milano, Milan, Italy; 6 Department of Radiology, Lausanne University Hospital and University of Lausanne, Lausanne, Switzerland; 7 Department of Radiology, Freiburg University Medical Center, Freiburg, Germany; 8 Department of Radiology and Nuclear Medicine, Amsterdam UMC Location University of Amsterdam, Amsterdam, The Netherlands; 9 Department of Radiation Oncology, University of Groningen, University Medical Center Groningen, Groningen, The Netherlands

**Keywords:** Artificial intelligence, Radiomics, Sarcoma, Texture analysis

## Abstract

**Objective:**

To systematically review radiomic feature reproducibility and model validation strategies in recent studies dealing with CT and MRI radiomics of bone and soft-tissue sarcomas, thus updating a previous version of this review which included studies published up to 2020.

**Methods:**

A literature search was conducted on EMBASE and PubMed databases for papers published between January 2021 and March 2023. Data regarding radiomic feature reproducibility and model validation strategies were extracted and analyzed.

**Results:**

Out of 201 identified papers, 55 were included. They dealt with radiomics of bone (*n *= 23) or soft-tissue (*n* = 32) tumors. Thirty-two (out of 54 employing manual or semiautomatic segmentation, 59%) studies included a feature reproducibility analysis. Reproducibility was assessed based on intra/interobserver segmentation variability in 30 (55%) and geometrical transformations of the region of interest in 2 (4%) studies. At least one machine learning validation technique was used for model development in 34 (62%) papers, and K-fold cross-validation was employed most frequently. A clinical validation of the model was reported in 38 (69%) papers. It was performed using a separate dataset from the primary institution (internal test) in 22 (40%), an independent dataset from another institution (external test) in 14 (25%) and both in 2 (4%) studies.

**Conclusions:**

Compared to papers published up to 2020, a clear improvement was noted with almost double publications reporting methodological aspects related to reproducibility and validation. Larger multicenter investigations including external clinical validation and the publication of databases in open-access repositories could further improve methodology and bring radiomics from a research area to the clinical stage.

**Critical relevance statement:**

An improvement in feature reproducibility and model validation strategies has been shown in this updated systematic review on radiomics of bone and soft-tissue sarcomas, highlighting efforts to enhance methodology and bring radiomics from a research area to the clinical stage.

**Key points:**

• 2021–2023 radiomic studies on CT and MRI of musculoskeletal sarcomas were reviewed.

• Feature reproducibility was assessed in more than half (59%) of the studies.

• Model clinical validation was performed in 69% of the studies.

• Internal (44%) and/or external (29%) test datasets were employed for clinical validation.

**Graphical Abstract:**

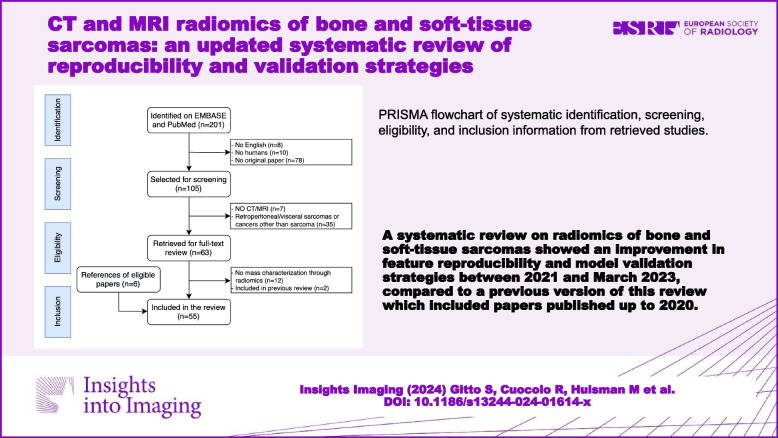

**Supplementary Information:**

The online version contains supplementary material available at 10.1186/s13244-024-01614-x.

## Introduction

The term “radiomics” indicates the extraction and analysis of large amounts of quantitative parameters, also known as radiomic features, from medical images [1]. Similar to other “omics” technologies (e.g., genomics and proteomics), the extraction of quantitative information from images obtained during standard clinical workflows may potentially enable an extensive tumor characterization, including its genotype and predictions regarding prognosis [1–3]. Although radiomics holds great potential to augment clinical decision-making, translation to clinical practice is very limited compared to preclinical software development [4, 5]. The translational gap is at least partially attributable to low overall methodological quality of radiomics research and reporting. This was recently highlighted in a systematic review evaluating the application of the Radiomics Quality Score [6], which was proposed by Lambin et al. in 2017 and is currently the most widespread tool to assess the comprehensiveness and adequacy of radiomic pipelines, as well as the quality of their reporting [7]. Another important initiative aiming to improve standardization and reproducibility was the Image Biomarker Standardization Initiative, which provided a stepwise consensus for different parts of execution of radiomics pipelines [8].

To bridge the gap between academic endeavors and real-life application, certain challenges of radiomics must be addressed carefully. As radiomics is based on a two-step approach consisting of data extraction and analysis [9], the main challenge of the first step (i.e., data extraction) is the reproducibility of radiomic features, which is influenced by several parameters related to image acquisition, region of interest (ROI) delineation and post-processing [10, 11]. The main challenge of the second step (i.e., data analysis) is validation of the radiomics-based models, which are built with the aim of predicting the diagnosis or outcome of interest [11]. The issues of feature reproducibility and validation strategies are well addressed as separate items in Radiomics Quality Score [7]. Additionally, they are included in international guidelines recently published to guide the translation of radiomics into clinical practice, such as criteria for development of radiomic models [12] and a checklist for evaluation of radiomics research endorsed by the European Society of Radiology and European Society of Medical Imaging Informatics [13].

In musculoskeletal oncology, radiomic studies have shown encouraging results to improve diagnosis and prognosis prediction of bone and soft-tissue sarcomas [14], which are rare cancers where quantitative imaging data may certainly aid in clinical management. Reproducibility and validation strategies in radiomics of bone and soft-tissue sarcomas were assessed in a previous systematic review including papers published up to December 2020 [14]. Reproducibility analysis and independent clinical validation were reported in 37% and 10% of the papers, respectively [14]. Particularly, the relative rarity of bone and soft-tissue sarcomas certainly contributed to preventing model validation in large datasets, thus highlighting the need for multi-center investigations or registries. Hence, the authors recommended future efforts to bring the field of radiomics from a preclinical research area to the clinical stage [14]. Since then, the number of radiomics research papers has rapidly increased. Combined with the great attention currently paid to reproducibility and validation strategies in radiomic workflows, this increase highlights the need for an update of the previous review [14] following guidelines on when and how to update systematic reviews [15]. Thus, the aim of our current study is to systematically review radiomic feature reproducibility and model validation strategies in recent studies dealing with computed tomography (CT) and magnetic resonance imaging (MRI) radiomics of bone and soft-tissue sarcomas, which have been published since 2021. The ultimate goal is to promote and facilitate a consensus on feature reproducibility and model validation in radiomic workflows.

## Methods

The study was registered on the International Prospective Register of Systematic Reviews database with the registration number CRD42023395542. The methods used in the current review paralleled those employed in the previous version [14], except for the number of reviewers involved in literature search, study selection, and data extraction, namely three in the current and two in the previous reviews. Additionally, in data extraction, segmentation process and style were grouped under baseline study characteristics in the previous review [14]. Conversely, these items constituted a separate category in the current version, which also included information regarding radiomic feature types as broad categories.

### Reviewers

Literature search, study selection, and data extraction were performed independently by three musculoskeletal radiologists with 3 to 5 years of experience in radiomics and bone and soft-tissue sarcomas (S.G., C.M., D.A.). In case of disagreement, an agreement was achieved by consensus of these three readers and a fourth radiologist with 8 years of experience in artificial intelligence and radiomics (R.C.). The Preferred Reporting Items for Systematic reviews and Meta-Analyses (PRISMA) guidelines were followed [16]. PRISMA checklist is provided as a supplementary table (Supplementary file 1).

### Search strategy

An electronic literature search was conducted on EMBASE (Elsevier) and PubMed (MEDLINE, US National Library of Medicine and National Institutes of Health) databases for studies dealing with CT and MRI radiomics of bone and soft-tissue sarcomas, which were published between 1^st^ January 2021 and 31^st^ March 2023. A controlled vocabulary was adopted using medical subject headings in PubMed and the thesaurus in EMBASE. Search syntax was built by combining search terms related to two main domains, namely “musculoskeletal sarcomas” and “radiomics.” The exact search query was: (“sarcoma”/exp OR “sarcoma”) AND (“radiomics”/exp OR “radiomics” OR “texture”/exp OR “texture”). Studies were first screened by title and abstract. The full text and supplementary material of eligible studies were retrieved for further review. The references of eligible papers were also checked for additional publications to include.

### Inclusion and exclusion criteria

Inclusion criteria were (i) original research studies published in peer-reviewed journals; (ii) focus on CT or MRI radiomics-based characterization of sarcomas located in bone and soft tissues for either diagnosis- or prognosis-related tasks; (iii) statement that local ethics committee approval was obtained, or ethical standards of the institutional or national research committee were followed. Exclusion criteria were (i) studies not dealing with mass characterization, such as those focused on computer-assisted diagnosis and detection systems; (ii) studies concerning retroperitoneal and visceral sarcomas or cancers other than sarcoma; (iii) animal, cadaveric or laboratory studies; (iv) papers published in languages other than English; (v) studies already included in the previous version of this review [14], such as those published online in 2020 and in a volume/issue in 2021.

### Data extraction

Data were extracted to a spreadsheet with a drop-down list for all items, which were grouped into four main categories, namely baseline study characteristics, segmentation and radiomic feature type, radiomic feature reproducibility strategies, and predictive model validation strategies. Items regarding baseline study characteristics included first author’s last name, year of publication, study aim, tumor type, study design, reference standard, imaging modality, database size, and use of public data. Items concerning segmentation and radiomic feature types were segmentation process, segmentation style, and radiomic feature types as broad categories. Items regarding radiomic feature reproducibility included strategies, statistical methods, and thresholds used for reproducibility analysis. Finally, items concerning model validation included the use of machine learning validation techniques, clinical validation performed on a separate internal dataset, and clinical validation performed on an external dataset.

## Results

### Baseline study characteristics

**Fig. 1:**
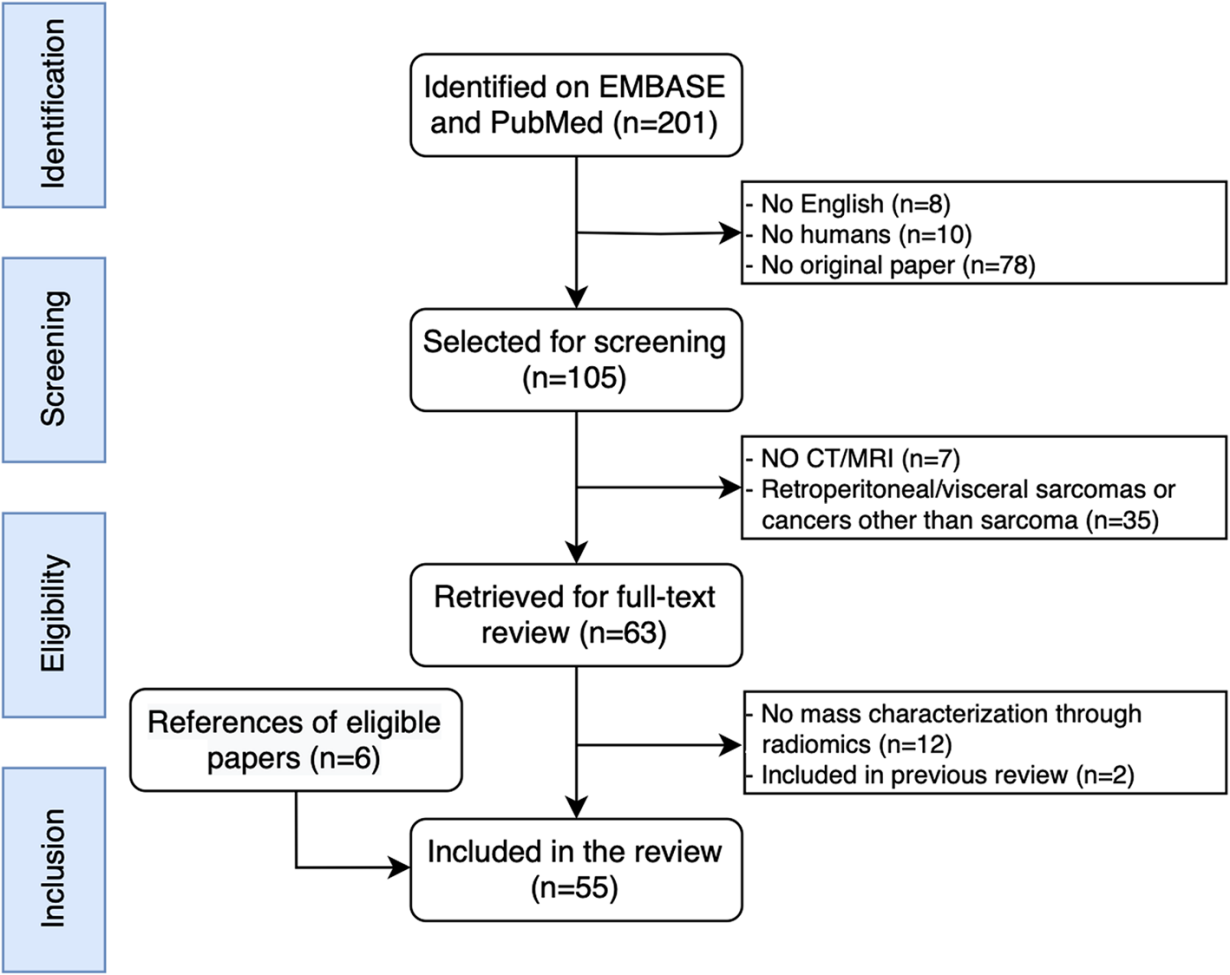
PRISMA (Preferred Reporting Items for Systematic reviews and Meta-Analyses) flowchart of systematic identification, screening, eligibility, and inclusion information from retrieved studies

A flowchart showing the literature search process is shown in Fig. 1. After screening 201 papers and applying the eligibility criteria, 55 papers were finally included in this systematic review. Tables 1 and 2 show the characteristics of studies on radiomics of bone (*n *= 23) and soft-tissue (*n *= 32) sarcomas, respectively.

**Table 1:** Characteristics of the included studies on bone sarcomas

First author	Year	Aim	Tumor type	Design	Reference standard	Imaging method	Database (*n*)	Public data	Segmentation		Feature type
									Process	Style	
Bouhamama [64]	2022	Therapy response	Osteosarcoma	Retrospective	Histology	MRI	176	No	Manual	3D	Handcrafted
Buizza [24]	2021	Survival Therapy response	Chordoma	Retrospective	Clinical/imaging follow-up	CT MRI	57	No	Manual	3D	Handcrafted
Chen [44]	2021	Therapy response	Osteosarcoma	Retrospective	Histology	MRI	102	No	Manual	3D	Handcrafted
Chianca [22]	2021	Benign vs malignant	Different benign/malignant histotypes	Retrospective	Histology Imaging follow-up (benign)	MRI	146	No	Manual	2D without MS	Handcrafted
Cilengir [20]	2023	Benign vs malignant	Enchondroma Chondrosarcoma	Retrospective	Histology Imaging follow-up (benign)	MRI	47	No	Manual	3D	Handcrafted
Deng [70]	2021	Benign vs malignant Grading	Enchondroma Chondrosarcoma	Retrospective	Histology	CT MRI	91	No	Manual	2D without MS	Handcrafted
Gitto [38]	2022	Grading	Chondrosarcoma	Retrospective	Histology	MRI	158	No	Manual	2D without MS	Handcrafted
Gitto [49]	2021	Grading	Chondrosarcoma	Retrospective	Histology	CT	120	No	Manual	2D without MS	Handcrafted
Gitto [57]	2022	Therapy response	Ewing sarcoma	Retrospective	Histology	MRI	30	No	Manual	3D 2D without MS	Handcrafted
Gitto [58]	2022	Benign vs malignant	Different benign/malignant histotypes	Retrospective	Histology	MRI	101	No	Manual	3D	Handcrafted
Li [53]	2023	Grading	Chondrosarcoma	Retrospective	Histology	MRI	102	No	Manual	3D	Handcrafted
Liu [51]	2021	Local/metastatic relapse	Osteosarcoma	Retrospective	Histology Imaging follow-up	CT	80	No	Manual	3D	Handcrafted
Luo [37]	2022	Metastatic relapse	Osteosarcoma	Retrospective	Histology Imaging follow-up	MRI	78	No	Manual	3D	Handcrafted
Pan [45]	2021	Benign vs malignant	Enchondroma Chondrosarcoma	Retrospective	Histology	MRI	103	No	Manual	3D	Handcrafted
Pereira [47]	2021	Metastatic relapse	Osteosarcoma	Retrospective	Imaging follow-up	CT	81	No	Semiautomatic	3D	Handcrafted
Sun [41]	2021	Benign vs malignant	Different benign/malignant histotypes	Retrospective	Histology	CT	206	No	Manual	3D	Handcrafted
White [54]	2023	Survival Therapy response	Osteosarcoma	Retrospective	Clinical follow-up (survival) Histology (therapy response)	MRI	105	No	Manual	2D without MS	Handcrafted
Xu [28]	2021	Therapy response	Osteosarcoma	Retrospective	Histology	CT	157	No	Manual	3D	Handcrafted
Yamazawa [69]	2022	Tumor histotype	Chordoma Chondrosarcoma	Retrospective	Histology	MRI	57	No	Manual	3D	Handcrafted
Yin [65]	2021	Therapy complication	Different benign/malignant histotypes	Retrospective	Clinical/surgical data	CT	810	No	Semiautomatic	3D	Handcrafted
Yin [66]	2021	Tumor histotype	Different benign/malignant histotypes	Retrospective	Histology	CT	795	No	Semiautomatic	3D	Handcrafted
Zhang [42]	2021	Therapy response	Osteosarcoma	Retrospective	Histology	MRI	102	No	Manual	3D	Handcrafted
Zhong [31]	2022	Therapy response	Osteosarcoma	Retrospective	Histology	MRI	144	No	Automatic	2D without MS	Handcrafted

*MS *Multiple sampling

**Table 2:** Characteristics of the included studies on soft-tissue sarcomas

First author	Year	Aim	Tumor type	Design	Reference standard	Imaging method	Database (*n*)	Public data	Segmentation		Feature type
									Process	Style	
Cao [62]	2023	Benign vs malignant	Nerve sheath tumors	Retrospective	Histology	MRI	119	No	Manual	3D	Handcrafted Deep
Cay [34]	2022	Benign vs malignant	Lipoma ALT/WDLS	Retrospective	Histology	MRI	65	No	Manual	2D with MS	Handcrafted
Chang [30]	2022	Benign vs malignant	Different benign/malignant histotypes	Retrospective	Histology	MRI	39	No	Manual	2D without MS	Handcrafted
Chen [46]	2021	Survival	Different sarcoma histotypes	Retrospective	Clinical follow-up	MRI	62	Yes	Manual	3D	Handcrafted
Crombé [25]	2023	Natural evolution	Different sarcoma histotypes	Retrospective	RNA sequencing	MRI	63	No	Manual	3D	Handcrafted
Fadli [35]	2022	Local/metastatic relapse Survival	Different sarcoma histotypes	Retrospective	Histology (relapse) Clinical follow-up (survival)	MRI	63	No	Manual	3D	Handcrafted
Fields [18]	2021	Benign vs malignant	Different benign/malignant histotypes	Retrospective	Histology	MRI	128	No	Manual	3D	Handcrafted
Fields [27]	2023	Therapy response	Different sarcoma histotypes	Retrospective	Histology	MRI	44	No	Manual	3D	Handcrafted
Fradet [63]	2022	Benign vs malignant	Lipoma ALT/WDLS	Retrospective	Histology	MRI	145	No	Manual	3D	Handcrafted Deep
Giraudo [23]	2022	Grading Metastatic relapse Survival	Different sarcoma histotypes	Retrospective	Histology (grading) Clinical/imaging follow-up (outcome)	MRI	36	No	Manual	3D	Handcrafted
González-Viguera [80]	2021	Local/metastatic relapse Survival	Different sarcoma histotypes	Retrospective	Clinical/imaging follow-up	CT	25	No	Manual	3D	Handcrafted
Hu [19]	2021	Benign vs malignant	Different benign/malignant histotypes	Retrospective	Histology	MRI	161	No	Manual	3D	Handcrafted
Hu [33]	2022	Metastatic relapse	Different sarcoma histotypes	Retrospective	Histology	MRI	154	No	Manual	3D	Handcrafted
Lee [60]	2023	Marginal infiltration	Different sarcoma histotypes	Retrospective	Histology	MRI	72	No	Semiautomatic	3D	Handcrafted
Lee [61]	2021	Benign vs malignant	Different benign/malignant histotypes	Retrospective	Histology	MRI	151	No	Semiautomatic	3D	Handcrafted
Liang [36]	2022	Metastatic relapse	Different sarcoma histotypes	Retrospective	Histology Imaging follow-up	MRI	242	No	Manual	3D	Handcrafted Deep
Liu [26]	2022	Local relapse	Different sarcoma histotypes	Retrospective	Histology Imaging follow-up	MRI	282	No	Manual (handcrafted) None (deep)	3D	Handcrafted Deep
Miao [40]	2022	Therapy response	Different sarcoma histotypes	Prospective	Histology	MRI	30	No	Manual	3D	Handcrafted
Navarro [67]	2021	Grading	Different sarcoma histotypes	Retrospective	Histology	MRI	306	No	Manual	3D	Deep
Ozturk [21]	2021	Benign vs malignant	Different benign/malignant histotypes	Retrospective	Histology Imaging follow-up (benign)	MRI	53	No	Manual	3D	Handcrafted
Peeken [43]	2021	Therapy response	Different sarcoma histotypes	Retrospective	Histology	MRI	156	No	Manual	3D	Handcrafted
Peeken [50]	2021	Survival	Different sarcoma histotypes	Retrospective	Clinical follow-up	MRI	179	No	Manual	3D	Handcrafted
Ristow [17]	2022	Benign vs malignant	Nerve sheath tumors	Retrospective	Histology Imaging follow-up (benign)	MRI	142	No	Manual	3D	Handcrafted
Sudjai [52]	2023	Benign vs malignant	Lipoma ALT/WDLS	Retrospective	Histology	MRI	68	No	Manual	3D	Handcrafted
Tang [59]	2022	Benign vs malignant	Lipoma ALT/WDLS	Retrospective	Histology	MRI	122	No	Manual	3D	Handcrafted
Yan [48]	2021	Grading Survival	Different sarcoma histotypes	Retrospective	Histology (grading) Clinical/imaging follow-up (survival)	MRI	180	No	Manual	3D	Handcrafted
Yang [29]	2022	Survival	Sarcoma NOS	Retrospective	Clinical follow-up	CT	353	No	Manual	Not specified	Handcrafted
Yang [39]	2022	Proliferation index	Sarcoma NOS	Retrospective	Histology	MRI	149	No	Manual	3D	Handcrafted
Yang [55]	2022	Grading Survival	Different sarcoma histotypes	Retrospective	Histology (grading) Clinical follow-up (survival)	MRI	540	No	Manual (handcrafted) Automatic (deep)	3D	Handcrafted Deep
Yang [56]	2022	Benign vs malignant	Lipoma ALT/WDLS	Retrospective	Histology	CT MRI	127	No	Manual	3D	Handcrafted Deep
Yue [32]	2022	Benign vs malignant	Different benign/malignant histotypes	Retrospective	Histology	MRI	148	No	Manual	3D	Handcrafted
Zhang [68]	2021	Benign vs malignant	Nerve sheath tumors	Retrospective	Histology	MRI	266	No	Manual	3D	Handcrafted

*MS *Multiple sampling; *ALT/WDLS *Atypical lipomatous tumor/well-differentiated liposarcoma

Twenty-four out of 55 studies (44%) were published in 2021, 23 (42%) in 2022, and 8 (14%) between January and March 2023. The design was prospective in 1 study (2%) and retrospective in the remaining 54 studies (98%). The investigated imaging modality was MRI (one or multiple sequences) in 43 studies (78%), CT in 9 (16%), and a combination of both in 3 (6%). The median size of the database was 120 lesions (range 25–810). In 3 studies multiple lesions for the same patient(s) were considered, thus including 142 [17], 128 [18], and 161 [19] lesions from 36, 125, and 160 patients, respectively. Public data were used only in 1 (2%) study.

Included studies aimed at predicting either diagnosis or prognosis. In diagnostic studies, classification tasks were benign vs. malignant (including intermediate malignancies such as atypical lipomatous tumor) tumor discrimination (*n* = 20), grading (*n *= 8), tumor histotype discrimination (*n *= 2), proliferation index Ki-67 expression (*n *= 1), and evaluation of marginal infiltration (*n *= 1). Prognostic studies aimed at predicting survival (*n *= 10), local and/or metastatic relapse (*n *= 9), response to chemotherapy or radiotherapy (*n *= 11), treatment complications (*n *= 1), and natural evolution over time before starting any treatment (*n *= 1). It should be noted that the aim was two- or threefold in some studies, as detailed in Tables 1 and 2. In studies focused on diagnosis-related tasks, histology was the reference standard in all cases except benign lesions diagnosed on the basis of stable imaging findings over time in four papers [17, 20–22]. In studies dealing with survival prediction, survival was assessed based on clinical follow-up. In studies focused on the prediction of tumor relapse, the reference standard was based on histology or clinical and imaging follow-up. In one study, the criteria for determining relapse were not specified [23]. In studies aimed at therapy response prediction, the reference standard was histology in all but one study where the response was assessed based on clinical and imaging evaluation [24]. Treatment complications were assessed based on clinical and surgical data. In the study dealing with natural evolution monitoring, radiomics was correlated to gene expression assessed using RNA sequencing [25].

### Segmentation and feature types

The segmentation process was performed only manually in 48 (87%) studies, semiautomatically in 5 (9%) studies, both manually and automatically (for handcrafted and deep features, respectively) in 1 study (2%), and only automatically in 1 (2%) study. Of note, in one study, manual segmentation was performed to extract handcrafted features and, in parallel, deep features were extracted from the whole images with no segmentation [26]. In three studies, tumor borders were manually delineated on one image of interest, and ROIs were then co-registered with a different MRI sequence or imaging modality [18, 24, 27]. In another study, manual segmentation was performed to include the tumor area, and an additional cubic ROI was placed in a non-tumorous area to evaluate non-tumorous radiomics [28].

The following segmentation styles were identified: 3D in 45 (82%) studies, 2D without multiple sampling in 7 (13%) studies, 2D with multiple sampling in 1 (2%) study, and multiple segmentation styles such as 3D and 2D without multiple sampling in 1 (2%) study. In the remaining study, the segmentation style was not specified [29]. Of note, a single slice showing maximum tumor extension was chosen in all studies employing 2D segmentation without multiple sampling, except in one case where it was chosen based on tumor characteristics [30] and another study where the criteria for slice selection were not specified [31].

Regarding the radiomic feature types, 48 (87%) studies included only handcrafted features, 6 (11%) studies included both handcrafted and deep features, and the remaining (2%) study included only deep features.

### Feature reproducibility

**Fig. 2:**
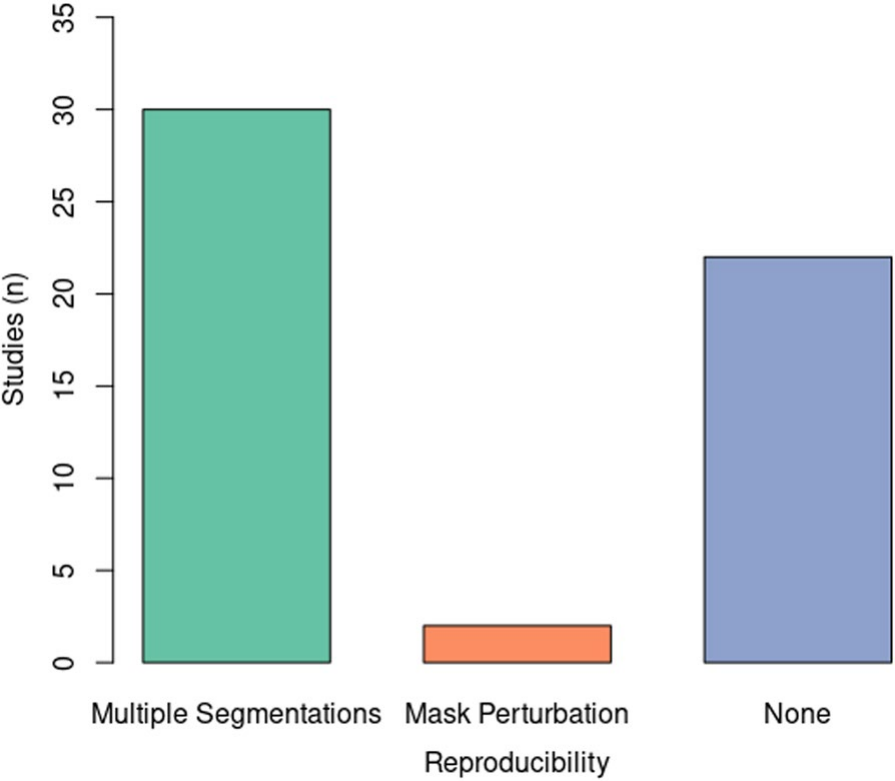
Bar plot showing the distribution of the employed feature reproducibility strategies among the included studies

Thirty-two (59%) of the 54 studies employing manual or semiautomatic segmentation process included a reproducibility analysis in their workflow. In 30 (55%) investigations [19–21, 23, 26, 32–56], the reproducibility of radiomic features was assessed based on repeated segmentations performed by different readers and/or the same reader at different time points. In 2 (4%) studies [57, 58], feature reproducibility was assessed through small geometrical transformations of the ROIs mimicking multiple manual delineations. In detail, small translations of the ROI were applied in different directions, and the entity of these translations was 10% of the length of the bounding box including the tumor [57, 58]. No studies evaluated feature reproducibility based on different acquisition or post-processing techniques. The distribution of the employed feature reproducibility strategies among the included studies is shown in the bar plot in Fig. 2. Of note, in 3 studies [59–61], repeated segmentations were performed to assess similarity (using Dice similarity coefficient) but feature reproducibility was not evaluated. Additionally, segmentations were validated by a second experienced reader in 7 studies [17, 25, 28–30, 62, 63] without, however, addressing the issue of feature reproducibility.

The intraclass correlation coefficient (ICC) was the statistical method used in all papers reporting a reproducibility analysis. ICC threshold ranged between 0.7 [54] and 0.9 [20, 46] for reproducible features. Additionally, the following statistical methods were used less commonly: Bland–Altman method [54], Pearson’s correlation coefficient [52], and Spearman’s rank-order coefficient [52].

### Validation techniques

**Fig. 3:**
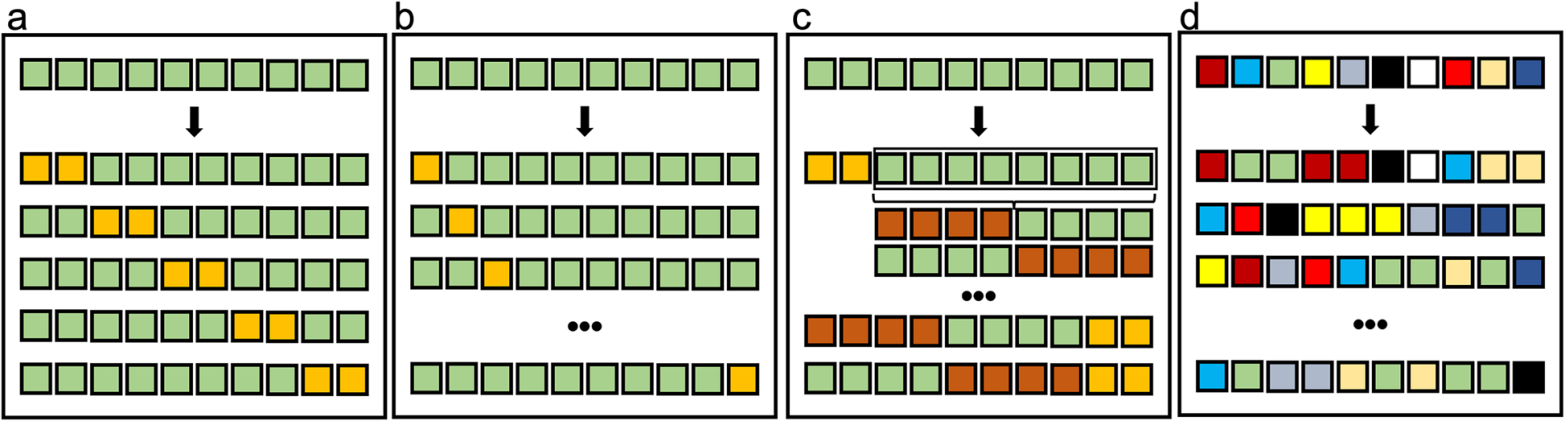
Overview of machine learning validation techniques. In k-fold cross-validation (**a**), the data is split into k equally sized partitions, and each is used in turn to validate a model trained on the remaining. The process for leave-one-out cross-validation (**b**) is the same, but k equals the total sample size. In nested cross-validation (**c**), an outer and an inner loops of k-fold cross-validation are performed. Typically, the inner loop is used for model tuning, and the outer one to assess its accuracy. Bootstrapping (**d**) is based on a different principle: random sampling from the original dataset is performed, with replacement. As a result, the produced samples may include multiple (or even no) instances of each original case

At least one machine learning validation technique was used in 34 (62%) of the 55 papers. K-fold cross-validation was used in most of the studies [18, 20, 22, 24, 27, 31, 37–39, 44, 47, 49, 52, 54, 57, 58, 60–68]. The following machine learning validation techniques were used less commonly: bootstrapping [34, 46], leave-one-out cross-validation [17, 28], and nested cross-validation [43, 55, 56, 69]. In one study, both K-fold cross-validation and nested cross-validation techniques were employed [50]. Figure 3 provides an overview of these machine learning validation techniques.

### Clinical validation

**Fig. 4:**
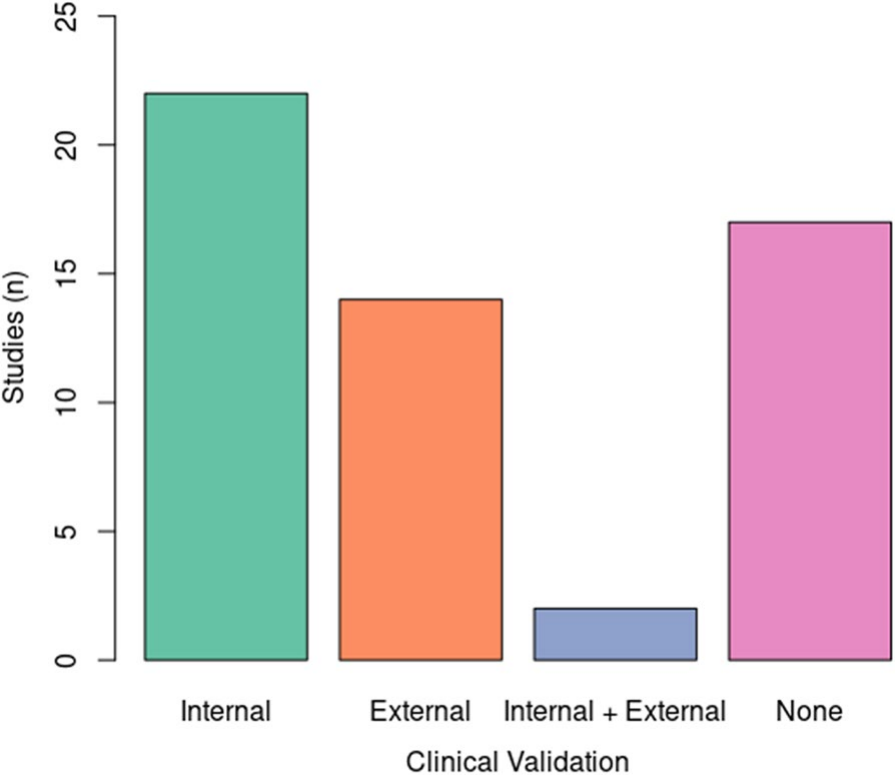
Bar plot showing the distribution of the employed clinical validation strategies among the included studies

A clinical validation of the radiomics-based prediction model was reported in 38 (69%) of the 55 studies. In 22 (40%) studies, it was performed on a separate set of data from the primary institution, namely the internal test dataset, which was chosen randomly [19, 20, 24, 28, 29, 31, 32, 37, 41, 42, 45, 47, 52, 53, 59, 65, 66, 68], based on temporal criteria [61, 69, 70] or different acquisition scanners [62]. Of note, in a multi-center study, patients were split into training and test cohorts randomly rather than following geographical criteria [68]. Thus, this was considered as an internal test dataset. In 14 (25%) studies [26, 36, 38, 39, 43, 44, 48–51, 56, 63, 64, 67], clinical validation was performed on an independent set of data from an external institution, namely the external test dataset. In 2 (4%) studies [22, 33], both internal and external test datasets were used for clinical validation. The distribution of the employed clinical validation strategies among the included studies is shown in the bar plot in Fig. 4. Radiomic feature reproducibility and model validation strategies of the included studies are summarized in Table 3, along with the same information extracted from the previous version of this review [14] for comparison.

**Table 3:** Radiomic feature reproducibility and model validation strategies of the studies included in the previous [14] and current review versions

	Previous review [14] (Studies published up to 2020)	Current review (Studies published between 2021 and March 2023)
**Reproducibility**		
Proportion of studies evaluating feature reproducibility	37% (18/49)	59% (32/54 studies employing manual or semiautomatic segmentation)
Strategies used for reproducibility assessment	Different acquisition techniques Different post-processing techniques Repeated segmentations performed by different readers or the same reader at different time points (mostly employed)	Repeated segmentations performed by different readers or the same reader at different time points (mostly employed) ROI geometrical transformations
Statistical methods	Analysis of variance Cronbach alpha statistic ICC (mostly employed) Pearson correlation coefficient Spearman correlation coefficient	Bland–Altman method ICC (mostly employed) Pearson’s correlation coefficient Spearman’s rank-order coefficient
**Validation**		
Proportion of studies employing machine learning validation techniques	51% (25/49)	62% (34/55)
Employed machine learning validation techniques	Bootstrapping K-fold cross-validation (mostly employed) Leave-one-out cross-validation Leave-p-out cross-validation Monte Carlo cross-validation Nested cross-validation Random-split cross-validation	Bootstrapping K-fold cross-validation (mostly employed) Leave-one-out cross-validation Nested cross-validation
Proportion of studies including clinical validation (internal or external)	39% (19/49)	69% (38/55)
Proportion of studies including internal clinical validation	29% (14/49)	44% (24/55, among which two studies also performed external clinical validation)
Proportion of studies including external clinical validation	10% (5/49)	29% (16/55, among which two studies also performed internal clinical validation)

## Discussion

This systematic review addressed the issues of feature reproducibility and validation strategies in CT and MRI radiomics of bone and soft-tissue sarcomas, as these are two main challenges hampering the generalizability of radiomic models and preventing their clinical implementation. Among papers published between January 2021 and March 2023, more than half reported a reproducibility analysis of radiomic features (59%) and a clinical validation of the predictive model against an internal test dataset, an external test dataset, or both (69% overall, among which 29% also or exclusively external). These assessments almost doubled compared to the previous version of this review including papers published up to December 2020, where they amounted to 37% and 39%, respectively [14]. Hence, although the percentage of investigations without any reproducibility and/or validation assessment is still considerable, significant efforts have been made to include them in radiomics studies to facilitate generalizability and thus clinical transferability. In particular, external clinical validation is crucial to ensure clinical translation of imaging biomarkers and should be encouraged.

CT and MRI radiomics of bone and soft-tissue sarcomas have progressively gained attention in musculoskeletal oncology to solve several diagnosis- or outcome-related tasks. In the previous version of this review [14], a rapid increase in research papers was observed and almost half of them (*n *= 23) were published in 2020. Since then, the number of new publications has remained almost unchanged every year, with 24 papers in 2021, 23 in 2022, and 8 in the first trimester of 2023. Most included studies (98%) were retrospective, similarly to the previous review [14]. Although prospective studies could provide the highest level of evidence supporting the clinical validity and usefulness of radiomic biomarkers [7], bone and soft-tissue sarcomas are low prevalent [71, 72] and retrospective design allows including relatively large amounts of data already available in radiology departments. The median size of the database was 120 lesions, having doubled compared to the previous review [14]. Of note, the use of public data was described only in one study dealing with soft-tissue sarcomas (2%) [46], even less than the previous review where it was reported in three cases [14]. Specifically, a public dataset available on The Cancer Imaging Archive was employed (https://www.cancerimagingarchive.net) [73]. Public datasets are essential to allow research groups from around the world to test and compare different radiomic models using common data. Hence, the use of public data should be promoted through new publicly available imaging databases in the future.

Segmentations included the entire tumor volume (3D) in most studies (84%) and, less frequently, single slices (2D) with or without multiple sampling. The segmentation process was performed manually in most studies (89%) and semiautomatically less frequently, as also observed in the previous review [14]. In addition, a fully automatic segmentation was used in two investigations (4%, one of which employing both automatic and manual segmentations). Furthermore, while most studies included only handcrafted features, deep features were employed in 13% of the studies (either alone or together with handcrafted features). In contrast to handcrafted features based on predefined mathematical formulas, deep features are obtained inside the layers of convolutional neural networks [74]. Future investigations focusing on deep features and convolutional neural networks with the use of very large datasets will better highlight the potential value of deep learning methods in radiomic workflows.

Radiomic feature reproducibility was evaluated in more than half of the studies (59%) employing manual or semiautomatic segmentation, which increased by approximately three-quarters compared to the previous version of this review [14]. This methodological assessment allows for identifying robust features and avoiding biases related to non-reliable, noisy features [75]. Inter- and intra-observer variability related to multiple ROI delineations by different readers or the same reader at different time points was the focus of reproducibility analysis in most studies. Less frequently, ROI perturbations obtained through geometrical transformations were used to mimic multiple delineations and evaluate feature reproducibility. No study assessed the influence of image acquisition parameters or post-processing techniques on feature reproducibility. Thus, this latter domain deserves further investigation, which could be facilitated by prospective design in future studies. Finally, ICC was the statistical method of choice in all studies including a reproducibility analysis, with threshold values ranging from 0.7 to 0.9, which were in line with recent guidelines for performing and assessing ICC [76].

At least one machine learning validation technique was used in more than half (62%) of the papers and K-fold cross-validation was performed most commonly, similarly to the previous review [14]. These resampling strategies are extremely useful with relatively limited data samples to reduce overfitting and better estimate the radiomic model performance on new data [77, 78]. Besides, a clinical validation of the radiomic model should be performed through real testing against unseen data [79]. We found that clinical validation was reported in 69% of studies. In detail, it was performed against unseen separate data from the primary institution (internal test dataset) and unseen independent data from a different institution (external test dataset) in 44% and 29% of the studies, respectively. Of note, two studies (4%) included both internal and external test datasets for clinical validation. The number of radiomic papers reporting clinical validation increased compared to the previous review [14] and, particularly, the number of those including an external test dataset tripled. Although the percentage of studies without any clinical validation is not negligible and future efforts are required, this may suggest that we are on the right track to bridge the gap between research concepts and clinical application in radiomics of bone and soft-tissue sarcomas.

Some limitations of this study need to be considered. First, this review focused on feature reproducibility and model validation strategies employed in bone and soft-tissue sarcoma studies to facilitate achieving a consensus on these aspects in radiomic workflows. However, this consensus has still to be reached. Second, this study is limited to a systematic review and no meta-analysis was performed, as radiomic papers dealing with bone and soft-tissue sarcomas are heterogenous in terms of objectives and subgroups of sarcoma with relatively small sample size per each objective and subgroup. Additionally, most studies assessed reproducibility as a feature-reduction method in radiomic pipelines based on an ICC threshold, without reporting ICC values for all features. Finally, in studies reporting a clinical validation, different metrics were used for model performance estimation. All these reasons prevented us from including reproducibility and validation methods in a meta-analysis.

Limitations notwithstanding, feature reproducibility and validation strategies were systematically reviewed in radiomic studies dealing with bone and soft-tissue sarcomas and published between January 2021 and March 2023. Compared to a previous review addressing the same issues in studies published up to December 2020 [14], a clear improvement was noted with almost double publications reporting methodological aspects related to reproducibility and validation. Larger investigations involving multiple institutions and the publication of new databases in freely available repositories should be promoted to further improve the methodology of radiomic studies and bring them a from preclinical research area to the clinical stage.

### Supplementary Information

**Additional file 1.** PRISMA 2020 Checklist.

## Data Availability

Data supporting the results can be obtained upon request to the corresponding author.

## Abbreviations

CT: Computed tomography
ICC: Intraclass correlation coefficient
MRI: Magnetic resonance imaging
ROI: Region of interest
PRISMA: Preferred Reporting Items for Systematic reviews and Meta-Analyses
